# Detection of Oropouche virus segment S in patients and in*Culex quinquefasciatus* in the state of Mato Grosso, Brazil

**DOI:** 10.1590/0074-02760150123

**Published:** 2015-09

**Authors:** Belgath Fernandes Cardoso, Otacília Pereira Serra, Letícia Borges da Silva Heinen, Nayara Zuchi, Victor Costa de Souza, Felipe Gomes Naveca, Marcelo Adriano Mendes dos Santos, Renata Dezengrini Slhessarenko

**Affiliations:** 1 Universidade Federal de Mato Grosso, Faculdade de Medicina, Programa de Pós-Graduação em Ciências da Saúde, Cuiabá, MT, Brasil; 2 Fundação Oswaldo Cruz, Instituto Leônidas e Maria Deane, Manaus, AM, Brasil; 3 Laboratório Central, Secretaria de Estado de Saúde, Cuiabá, MT, Brasil

**Keywords:** OROV, Orthobunyavirus, Mato Grosso, mosquitoes, phylogenetic analysis

## Abstract

This study aimed to investigate the circulation of Orthobunyavirus species in the state of Mato Grosso (MT) Brazil. During a dengue outbreak in 2011/2012, 529 serum samples were collected from patients with acute febrile illness with symptoms for up to five days and 387 pools of female* Culex quinquefasciatus *captured in 2013 were subjected to nested-reverse transcription-polymerase chain reaction for segment S of the Simbu serogroup followed by nucleotide sequencing and virus isolation in Vero cells. Patients (5/529; 0.9%) from Cuiabá (n = 3), Várzea Grande (n = 1) and Nova Mutum (n = 1) municipalities were positive for the S segment of Oropouche virus (OROV). Additionally, eight/387 *Cx. quinquefasciatus *pools were positive for the segment, with a minimum infection rate of 2.3. Phylogenetic analysis indicated that all the samples belong to the subgenotype Ia, presenting high homology with OROV strains obtained from humans and animals in the Brazilian Amazon. The present paper reports the first detection of an Orthobunyavirus, possibly OROV, in patients and in* Cx. quinquefasciatus* mosquitoes in MT. This finding reinforces the notion that arboviruses frequently reported in the Amazon Region circulate sporadically in MT during dengue outbreaks.

Arboviruses (arthropod-borne viruses) are widely dispersed in sylvatic tropical areas around the globe. However, some of these viruses have the ability to maintain urban transmission cycles involving humans as susceptible hosts and highly anthropophilic vector species, such as dengue virus (DENV), yellow fever virus (YFV), Chikungunya virus and Oropouche virus (OROV), producing large epidemics (Moreli & Costa 2013).

The Bunyaviridae family currently contains more than 350 species classified into five genera according to morphological, antigenic and molecular properties (Elliott 2014). The Bunyaviridae virion is spherical and enveloped, approximately 100 nm in diameter, containing three negative single-stranded genomic RNA segments (S, M and L) surrounded by helicoidal nucleocapsids. The S-RNA is the most conserved RNA segment of orthobunyaviruses. Due to this property, S-RNA is largely used for phylogenetic reconstruction, including for OROV isolates (Saeed et al. 2000, Nunes et al. 2005, Acrani et al. 2010, Vasconcelos et al. 2011, Hang et al. 2014).

The Orthobunyavirus genus comprises 170 viruses, which are assembled into 48 species in 19 recognised serogroups (Chowdhary et al. 2012,Gauci et al. 2015). The Simbu serogroup is the most important epidemiologically and includes 25 antigenically related viruses classified into seven complexes. These seven include Simbu, Manzanilla, Oropouche, Akabane, Sathuperi, Shamonda and Shuni, which have been reported in all continents (Yanase et al. 2012). The Oropouche complex includes the OROV, Jatobal, Iquitos, Leanyer, Oya and Thimiri viruses. Of these, only OROV circulation is recognised in humans in Brazil. OROV is the most frequent Orthobunyavirus in the Amazon Region. South American strains of the virus obtained from human cases registered in Peru, Panamá, Brazil and Trinidad are classified mainly as genotypes I, II and III (Saeed et al. 2000, Vasconcelos et al. 2011).

OROV is transmitted in a sylvatic cycle that potentially involves birds, sloths and primates as amplifying hosts and *Aedes (Oclerotatus) serratus*,*Coquillettidia venezuelensis *and* Culicoides *spp as vectors (Figueiredo 1999). In villages located in degraded forests and in urbanised areas near forests humans are believed to act as amplifying hosts and *Culicoides paraensis *biting midges are believed to be the most important vector. *Culex quinquefasciatus *has been considered a secondary urban anthropophilic vector (Vasconcelos et al. 1989, Pinheiro et al. 1997). Clinical manifestations develop after three-eight days of incubation; Oropouche fever lasts for two-seven days, might be accompanied by exanthema and, in rare cases by aseptic meningitis, which is more frequent in immunocompromised individuals and children. Clinical recurrence is observed in 56% of the cases (Pinheiro et al. 1981b,Bastos et al. 2012).

OROV was first isolated from a febrile patient in Vega de Oropouche, Trinidad & Tobago in 1955 and from a *Coquilletidia venezuelensis* mosquito pool in 1960 (Anderson et al. 1961) *.* In Brazil, OROV was first isolated from a sloth (*Bradypus tridactylus*) and from *Ochlerotatus serratus *mosquitoes in 1960 during the construction of the Belém-Brasília Highway in the northern region of the country (Pinheiro et al. 1962). The first epidemic involved 11 thousand individuals and was registered in 1961 in a city of the state of Pará (PA), northern Brazil. Serological studies indicate that at least 357,000 individuals were infected by OROV in the Brazilian Amazon between 1961-1996, especially in PA (Pinheiro et al. 1986). Epidemics were also registered in other states, including Amazonas (AM) (Borborema et al. 1982) and Amapá (AP) in 1980 (Pinheiro 1983), Maranhão (MA) and Tocantins in 1988 (Vasconcelos et al. 1989) and Rondônia (RO) in 1991 (Terzian et al. 2009). The last large epidemic was reported in 1996, involving PA, AM and Acre (AC) (Pinheiro et al. 1998).

In the Southeast and Central-West Regions of Brazil, OROV has also been reported. In Ribeirão Preto, state of São Paulo, OROV antibodies were detected in two urban residents (Figueiredo et al. 1986). In the state of Minas Gerais (MG), OROV was recovered from a primate (Nunes et al. 2005). Additionally, 0.7% of 1,201 inhabitants of cities from southern state of Goiás presented antibodies against OROV in 1973. The prevalence of antibodies in cities of the Amazon Region is generally up to 3% (Pinheiro et al. 1981b). Residents in two cities from PA affected by the Cuiabá-Santarém Highway, on the border with MT, presented anti-OROV IgM antibodies (Nunes et al. 2009). Recently, a nonhuman primate tested positive for OROV in a haemagglutination inhibition test in the Pantanal, state of Mato Grosso do Sul (MS) (Batista et al. 2012) and another exhibited a serological response to the virus in *Cerrado* of MS (Batista et al. 2013).

MT, located in Central-West Brazil, presents a tropical climate and particular ecological conditions, such as biodiversity and is distributed in sylvatic areas of the Amazon,*Cerrado* and Pantanal biomes, conditions that favour arbovirus circulation. Local urban areas are also susceptible to arbovirus circulation. The occurrence of the Mayaro and Saint Louis encephalitis (SLEV) viruses was recently reported during a dengue outbreak in Cuiabá, the capital and the largest city of MT (Zuchi et al. 2014, Heinen et al. 2015). Therefore, the aim of this study was to investigate the circulation of the Orthobunyavirus from the Simbu serogroup in patients with febrile illness and in *Cx. quinquefasciatus* mosquitoes captured in MT.

## SUBJECTS, MATERIALS AND METHODS

*Clinical samples and ethics statement* - In this study, serum samples from 529 patients with acute febrile illness persisting for up to five days from 17 cities of MT were obtained between October 2011-July 2012 in the Public Health Central Laboratory (LACEN-MT). All samples had been tested previously for DENV serotypes and YFV by virus isolation followed by immunofluorescence and molecular techniques.

Serum samples were stored at -80°C at the Laboratory of Virology of the School or Medicine of the Federal University of Mato Grosso (UFMT). The viral RNA was extracted (Qiamp viral RNA mini kit; Qiagen, Germany) and immediately converted into genus-specific cDNA. Nested-reverse transcription-polymerase chain reaction (RT-PCR) for the segment S of the Simbu serogroup of the Orthobunyavirus genus was performed (Moreli et al. 2002).

The procedures involving human samples were previously approved by the institutional review board of the Julio Muller University Hospital Ethical Committee on Research under the register 100/2011. All epidemiological data obtained through the Information System for Notifiable Diseases records and/or directly from the patients were handled anonymously and confidentially.

*Sampling of Culex mosquitoes in Cuiabá* - Because most of the patients included in the study are residents of the metropolitan area of Cuiabá, a parallel study was conducted with mosquitoes. Specimens of *Cx. quinquefasciatus *(n = 387) were captured between 01:00 pm-05:00 pm during the rainy season (January-April 2013) with Nasci aspirators and hand nets from three places in each of 200 censitary sectors that were randomly selected in Cuiabá. The mosquitoes remained in the laboratory for at least 12 h, receiving artificial feeding with sugar water until identification with a dichotomy key (Forattini 2002) and with a molecular approach using semi-nested-PCR for *Cx. quinquefasciatus* (Smith & Fonseca 2004). Pools containing one-35 mosquito specimens according to place of capture, genus, species and sex were stored at -80ºC. Only female pools were included in the present study.

Pools were macerated and diluted in RNAse-free phosphate-buffered solution; 400 μL of the supernatant was used for total RNA and total DNA extraction (Trizol; Invitrogen, USA) and cDNA was immediately synthesised with Superscript III (Invitrogen) following the manufacturer’s instructions. The extracted DNA was used for molecular confirmation of the mosquito species.

*Semi-nested-PCR for Culex species* - The protocol used for*Cx*. *quinquefasciatus* identification was performed in 78 pools that were identified as *Culex pipiens complex*and 102 of *Culex* spp according to Smith and Fonseca (2004) with few modifications. In the first reaction, the primers B1246s (0.2 µM) and F1475 (0.2 µM) were used with the following cycling conditions: 94ºC for 5 min, 35 cycles of 94ºC for 30 s, 55ºC for 30 s and 72ºC for 1 min and a final extension at 72ºC for 5 min. This PCR product was subjected to semi-nested-PCR using 1 ng of PCR product, the primers B1246s (0.2 µM) and ACEquin (0.8 µM) and cycling conditions as follows: 94ºC for 5 min, 35 cycles of 94ºC for 30 s, 57ºC for 30 s, 72ºC for 1 min and final extension of 72ºC for 5 min.

*Nested-PCR for segment S of orthobunyaviruses belonging to the Simbu serogroup* - The protocol described by Moreli et al. (2002) was used to amplify segment S from the genome of orthobunyaviruses (961 bp) with a few modifications. Briefly, cDNA (8 µL) was amplified with BUN-S primer and was then subjected to a PCR reaction containing 10x PCR buffer, MgCl_2 _(2 mM), dNTPs (0.2 mM), the primers BUN-S (+) (0.6 µM) and BUN-C (-) (0.6 µM), 1 U of DNA polymerase (LGC Biotecnologia, Brazil) and ultrapure water for 50 µL of reaction following the cycling conditions described by the authors. The second PCR reaction targets a 300-bp region of Simbu serogroup members (BS-S and BS-C primers). This reaction was performed using 2 µL of the product of the first reaction and the same concentrations of reagents and cycling conditions, with a final volume of 25 µL. cDNA from an OROV strain (BeAn19991) and no template were included as controls; precautions to avoid contamination were undertaken during procedures. The positive control was sequenced to rule out contamination.

*Nucleotide sequencing and phylogenetic analysis* - The sequencing was performed using POP-7^TM^ and an ABI 3130 DNA Sequencer. Approximately 10-40 ng of purified nested-PCR product (300 bp of segment S) was amplified following the BigDye Terminator v.3.1 Cycle Sequencing protocol. The sequences were initially filtered by applying a Phred score cut-off of ≤ 20 using the Sequencing Analysis (Applied Biosystems, v.5.3.1) software, a procedure that was kindly performed by the Leônidas e Maria Deane Institute, Oswaldo Cruz Foundation (Fiocruz) Amazônia. Only the filtered sequences were considered for contig assembly after trimming the low-quality ends. Geneious R6 (Biomatters, v.6.0.5) was used for this purpose. The contigs were compared with reference sequences through the nucleotide Basic Local Alignment Search Tool (BLASTn, GenBank, PubMed).

Phylogenetic analysis included several nucleotide sequences of segment S from OROV strains available from GenBank (PubMed, National Center for Biotechnology Information). After alignment with CLUSTALW and analysis using Molecular Evolutionary Genetics Analysis (MEGA v.5.05), the best model of nucleotide substitution was determined by jModelTest (v.2.2.6). A phylogenetic tree was generated using a region of the N protein of OROV with 1,000 bootstrap replicates. The evolutionary history was inferred using the neighbour-joining method with Tamura three-parameter distance model and the rate variation among sites was modelled with a gamma distribution (shape parameter = 1). Outgroups included the Buttonwillow, Faceys Paddock, Ingwavuma, Mermet, Aino, Tinaroo and Akabane orthobunyaviruses.

*Inoculation in cell culture* - Samples positive for OROV according to nested-RT-PCR and nucleotide sequencing were diluted 1:10 and inoculated into 24-well polystyrene plates containing Vero cells (ATCC CCL-81). The cells were cultivated in RPMI-1640 medium supplemented with 5% foetal bovine serum (FBS). After the incubation period (2 h) at 37ºC and 5% CO_2_, the inoculum was removed and the monolayer was washed with RPMI-1640 medium containing antimycotic agents and antibiotics. The culture medium was replaced and the cells were observed daily for seven-10 days. After this period, the monolayers were harvested for total RNA extraction (Trizol) followed by nested-RT-PCR and sequencing, as previously described. Three passages were performed with the supernatant to ensure viral amplification.

*Data analysis* - The minimum infection rate (MIR) was calculated with the formula (number of positive pools/total specimens tested) x 1,000, considering the total of *Cx. quinquefasciatus* specimens tested (3,425 mosquitoes). The geospatial data were analysed with ArcMap (ESri ArcGIS, v.9.3).

*Accessions *- Nucleotide sequences from the segment S of OROV obtained in the present study were deposited in GenBank and PubMed with the accessions KP310500-KP310507 (mosquito pools) and KP347671-KP347674; KP954633 (human samples).

## RESULTS

**Fig. 1:**
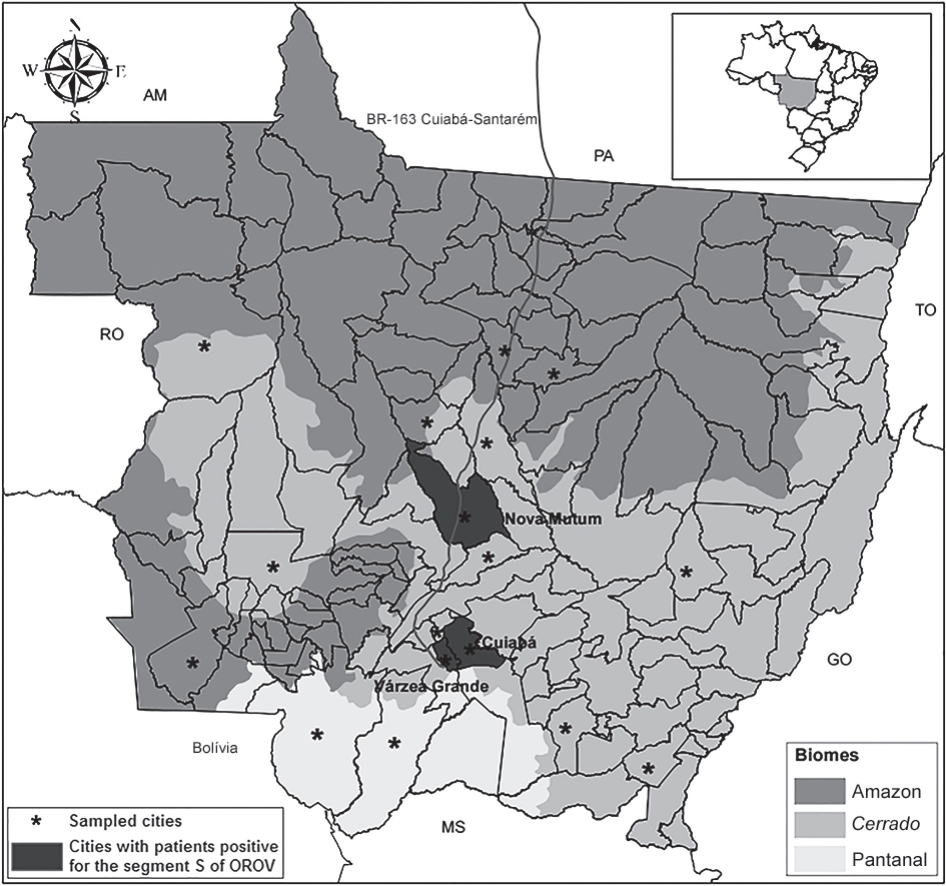
: distribution of the samples included in the study and the five patients with acute febrile illness positive for the segment S of Oropouche virus (OROV) by nested-reverse transcription-polymerase chain reaction per city of residency in the state of Mato Grosso, Brazil. Brazilian states: AM: Amazonas; GO: Goiás; MS: Mato Grosso do Sul; PA: Pará; RO: Rondônia; TO: Tocantins.

*Characterisation of the study population* - Among the 529 human samples included in the study, 461 (87.2%) were obtained from the metropolitan area of Cuiabá. In this subset, 396 (74.9%) were from Cuiabá, 65 (12.3%) were from Várzea Grande and 68 (12.8%) were from the other 15 cities of MT (Fig. 1). From this total, 255 (48.2%) were men, 263 (49.7%) were nonpregnant women and six (2.3%) were pregnant when the sample was collected. The age distribution consisted of 239 (45.3%) patients aged 20-39 years, 94 (17.8%) patients aged 40-59 years and 63 (12%) patients aged 15-19 years. Regarding the ancestral lineages, which were inferred by skin colour, 314 (59.4%) of the patients considered themselves *pardo* (a multiethnic group that represents over 42% of the total Brazilian population) (Lins et al. 2010). Additionally, 511 (96.6%) of the patients were residents in urban areas, 24 (4.6%) of the patients reported a recent history of travel or visit to rural or sylvatic areas and 267 (50.5%) of the patients denied any previous history of a similar disease.

*Detection of the segment S of OROV in serum samples of febrile patients from MT* - In this study, 5/529 (0.9%) serum samples of patients suspected of harbouring dengue who were treated at primary health care units were found to be positive for segment S of OROV according to nested-RT-PCR and subsequent nucleotide sequencing and virus isolation (Table I).

**TABLE I:** Clinical and epidemiologic characteristics of patients with acute febrile illness positive for the segment S of Oropouche virus (OROV)

Pacient ID [age (years)]	Sex	Skin colour	City of origin	Occupation	Clinical signs and symptoms and history of previous similar disease	Days of symptoms	Visit to rural/ sylvatic areas	RT-PCR	Virus isolation	GenBank accessions
101 (24)	M	*Pardo*	Várzea Grande	Autonomous	Myalgia, fever, headache, retroorbital pain, petechiae, pruritus	3	No	OROV/ DENV-4	P2	KP954633
282 (62)	F	White	Cuiabá	Retired	Hyperthermia, headache, myalgia, prostration and retroorbital pain - dengue history backwards	2	No	OROV	P3	KP347671
436 (24)	F	Black	Cuiabá	Student	Hyperthermia, headache, myalgia, prostration and retroorbital pain	2	Yes	OROV/ DENV-4	P2	KP347672
470 (29)	F	-	Nova Mutum	-	-	2	No	OROV	P3	KP347673
498 (14)	F	Black	Cuiabá	Student	Retroorbital pain, myalgia, nausea, arthralgia and prostration	2	No	OROV	P2	KP347674

DENV: dengue virus; F: female; M: male; P: passage in cell culture; RT-PCR: reverse transcription-polymerase chain reaction; -: not informed/available.

Four of the positive patients were residents of the metropolitan area of Cuiabá. All cities with positive patients are accessible by the Cuiabá-Santarém Highway (Fig. 1). Four of the patients were women and positive patients were identified only after two-three days of experiencing symptoms.

**Fig. 2:**
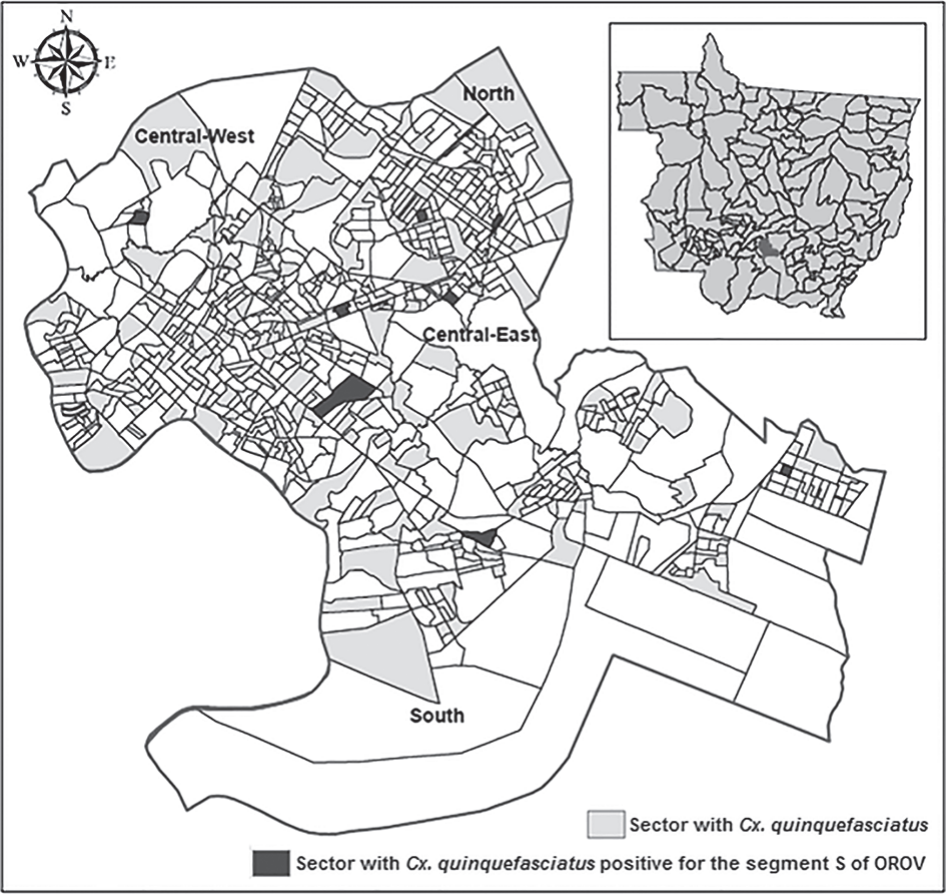
: distribution of *Culex quinquefasciatus* pools captured and positive for the segment S of Oropouche virus (OROV) according to the censitary map in Cuiabá, state of Mato Grosso, Brazil.

*Cx. quinquefasciatus captured in Cuiabá were positive for the segment S of OROV* - Among the 387 pools of* Cx. quinquefasciatus*, eight (2%) were positive for segment S of OROV by nested-RT-PCR and confirmed by nucleotide sequencing, with a MIR of 2.3 per 1,000 *Cx*.*quinquefasciatus* specimens. Although positive pools were identified in every month of sampling, 50% of the positive pools were captured in April 2013. Additionally, positive pools were identified in all of the administrative regions of the city (Fig. 2), three/eight of which were in the north (Table II).

**TABLE II:** *Culex quinquefasciatus* female pools captured in Cuiabá, state of Mato Grosso, Brazil between January-April 2013 positive for the segment S of Oropouche virus (OROV) and dengue virus 4 (DENV-4)

Pool ID	Engorged	Specimens (n)	Region	Capture date	RT-PCR	Virus isolation	GenBank accessions
116	Yes	34	Central-West	23 January	OROV	-	KP310505
549	Yes	1	Central-East	20 February	OROV/DENV-4	-	KP310504
632	No	2	Central-East	28 February	OROV	p3	KP310506
833	Yes	19	South	6 March	OROV/DENV-4	p2	KP310507
1229	No	2	South	9 April	OROV	-	KP310500
1256	No	2	North	10 April	OROV	-	KP310501
1458	No	1	North	22 April	OROV	p2	KP310502
1521	Yes	3	North	24 April	OROV	-	KP310503

p: passage in cell culture; RT-PCR: reverse transcription-polymerase chain reaction; -: negative through virus isolation.

**Fig. 3:**
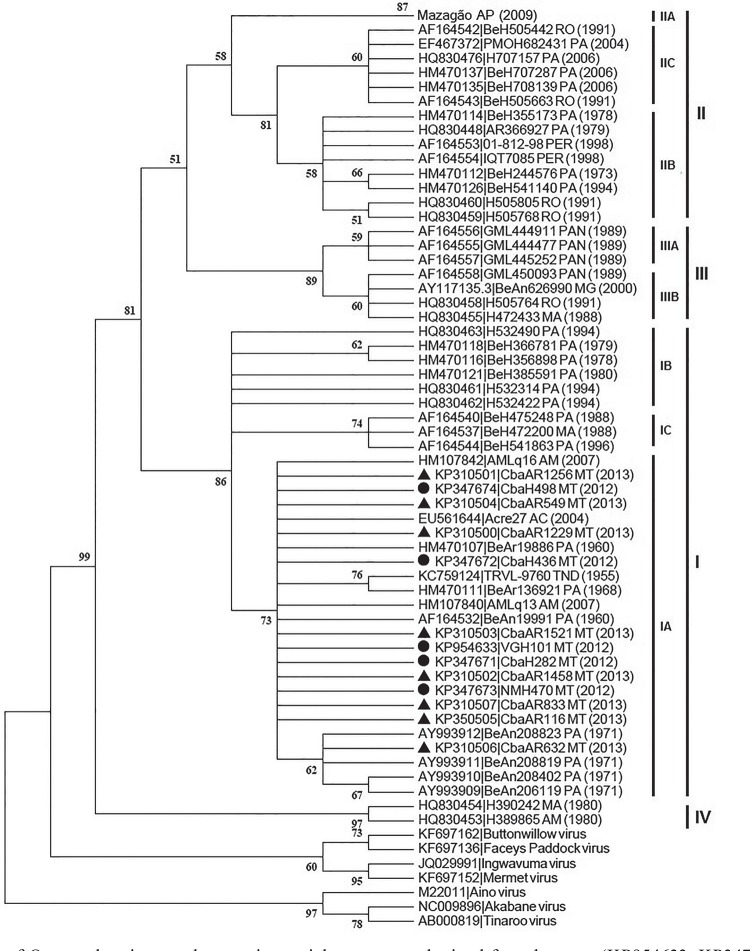
: phylogenetic tree of Oropouche virus nucleoprotein partial sequences obtained from humans (KP954633, KP347671-KP347674) and from*Culex quinquefasciatus* (KP310500-KP310507) in the state of Mato Grosso and reference strains from genotypes Ia-c, IIa-c, IIIa-b and IV of the virus outgroup: Buttonwillow (KF697162), Faceys Paddock (KF697136), Ingwavuma (JQ029991), Mermet (KF697152), Aino (M22011), Tinaroo (AB000819) and Akabane (NC009896) viruses. The tree was obtained through neighbour-joining method with bootstrap of 1,000 replicates. The distance was calculated by the transition/transversion rate, Tamura three-parameter method and gamma distribution (distribution parameter gamma = 1). ●: human samples; ▲: arthropod pools.

*Phylogenetic analysis of a partial region of the OROV nucleocapsid protein* - The phylogenetic analysis of the human (KP347671-KP347674; KP954633) and *Cx*. *quinquefasciatus*(KP310500-KP310507) samples positive for segment S of OROV indicate a close proximity between sequences of the virus belonging to subgenotype Ia (Fig. 3). The nucleotide similarity of the samples obtained in this study ranged from 98-100% for the sequences of OROV strains obtained from humans in Manaus, AM (AMLq16 and AMLq13), AC (BR/2004/Acre27) and from sloths (*B. tridactylus) *captured in PA (BeAn208823, BeAn208819, BeAn206119, BeAn208402).

Differences of zero-eight nucleotides were observed between segment S OROV sequences obtained from samples in the study. The highest divergence was verified between the sequences obtained from human samples 436, 498 and mosquito pool 632 (1.23 of distance). Human samples 282 of Cuiabá and 470 of Nova Mutum and all other sequences obtained from *Cx*. *quinquefasciatus* presented 100% identity.

**Fig. 4:**
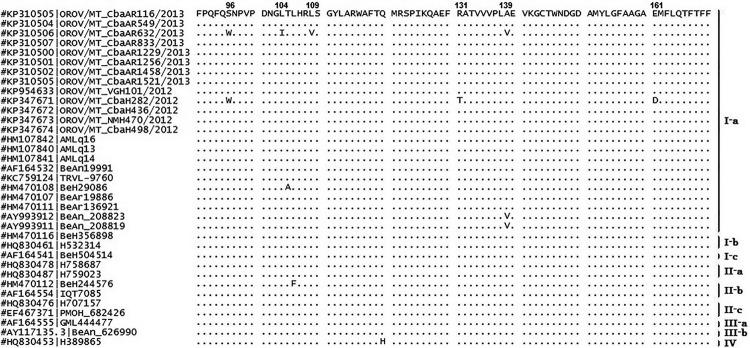
: alignment of the deduced amino acid (aa) nucleoprotein partial sequence of Oropouche virus (OROV) obtained from *Culex quinquefasciatus* pools (OROV/MT_CbaAR) and humans (OROV/MT_CbaH and OROV/MT_NMH) with acute febrile illness from the state of Mato Grosso, Brazil compared to reference strains available at GenBank database. Sites with aa substitutions are highlighted according to their position.

The sequence obtained from mosquito pool 632 presented the highest divergence of the nucleotide sequences obtained in the present study. At position 339, a silent cytosine-to-thymine mutation was observed, similar to that observed in OROV isolate sequences obtained in AP in 2009 belonging to genotype IIa. At position 418, a cytosine-to-thymine mutation resulted in an alanine-to-valine amino acid (aa) substitution at residue 139 (Fig. 4). This point mutation has been described only in animal strains belonging to subgenotype Ia obtained from sloths (*Bra-dypus trydactilus) *in PA (BeAn206119, BeAn208402, BeAn208819, BeAn208823). In sequences obtained from human samples 282 and 436, a guanine was substituted for an adenine at position 240; this substitution is commonly found in isolates belonging to subgenotype IIIb (BeAn626990, GML450093, H472433, H505764).

DENV-4 was identified in two patients (KP694222-KP694223) in previous studies and in two pools of mosquitoes (KP694224-KP694225) that were positive for both viruses (data not shown).

## DISCUSSION

Dengue outbreaks are reported every year in MT. Arboviruses represent a major issue for public health in tropical areas worldwide. There is evidence of OROV circulation in all neighbouring states, but no reports exist of OROV studies in MT.

Factors such as deforestation and new agricultural boundaries have contributed to the emergence of Oropouche fever in Brazil (Murphy 1998). It is estimated that more than 30 outbreaks have occurred in the country, constituting the second most frequent arbovirus in terms of number of cases reported to the Ministry of Health (Vasconcelos et al. 1989, Moreli et al. 2002). It is likely that the circulation of OROV and other orthobunyaviruses is underestimated in Brazil because the disease is clinically mistaken for dengue fever due to poor laboratorial differential diagnosis.

In this study, segment S of OROV was detected in five febrile patients from three cities located in the metropolitan area of Cuiabá and in northern MT during a dengue outbreak in 2012. The Cuiabá-Santarém Highway is accessed by all three cities. The central Amazon Region extends from northeast PA to the Porto Velho-Manaus-Venezuela Highway and is constantly evolving due to the soybean outflow (Becker 2001). A similar situation exists in the region surrounding the Cuiabá-Santarém Highway, which involves areas of*Cerrado* and Amazon and presents an intense flux of animals and persons, thereby propitiating arbovirus dispersion. The indiscriminate anthropic interference with sylvatic areas *via* deforestation and uncontrolled urbanisation also contributes to the large environmental impact and may result in the emergence or re-emergence of arboviruses and other viral diseases (Nunes et al. 2009).

Segment S of OROV was also identified in pools of female* Cx. quinquefasciatus* captured in Cuiabá during the rainy season of 2013. Although low in frequency, infection in humans (0.9%) and mosquitoes (MIR = 2.3 per 1,000 specimens) in the sampled populations indicates that OROV or other recombinant orthobunyavirus with the OROV segment S possibly circulate in the state. Consequently, outbreaks may take place under favourable conditions.

The different biotypes present in Cuiabá, are characterised by the existence of permanent preservation areas inside the urban perimeter, allowing the presence of host and vector species susceptible to arbovirus and, consequently, their occurrence in the human population. Lately, MT has undergone major environmental changes due to uncontrolled urban and agricultural expansion and the construction of new highways. Additionally, proximity to PA, RO and AM, where Oropouche fever cases are frequent, could have favoured the introduction OROV in MT. In this regard, IgM anti-OROV has been reported in residents of two cities from PA that border MT accessible*via* the Cuiabá-Santarém Highway (Nunes et al. 2009). This highway covers 1,780 km from Cuiabá to Santarém and is part of the BR-163 road, with 3,467 km of extension. This road crosses the country from the southern region through the coast of the Amazon River and the Trans-Amazonian Highway until Santarém. Anti-OROV serology in primates was also reported recently in MS (Batista et al. 2012, 2013).

Some authors have described a higher incidence of OROV infection in women (Dixon et al. 1981, Mourão et al. 2009). In this study, four of the five patients positive for segment S of OROV were women. Among the positive patients, only one reported a recent visit to rural areas. The other patients were urban residents without a history of travel or access to sylvatic or rural areas and were aged 14-62 years with different occupations, indicating the transmission may have occurred within the urban perimeter.

The positive patients were symptomatic for 48-72 h. Individuals with more than three days of symptoms probably were not detected due to low viraemic levels observed after the third day. For OROV, the reduction in viral titre is estimated at 72% on the third day and 44% and 23% on the fourth and fifth days, respectively, after the beginning of clinical signs, probably resulting in viraemic levels lower than the limit of detection by conventional diagnostic techniques (Pinheiro et al. 1981a).

The OROV nucleotide sequences of the S segment obtained from patients in MT present high identity with the S segment sequences of OROV strain AMLq16 obtained from the cerebrospinal fluid of one patient with aseptic meningitis in 2007 in Manaus (Bastos et al. 2012).

*Cx. quinquefasciatus* has been considered a secondary vector for OROV. Natural infection of *Cx. quinquefasciatus *by OROV captured in PA was demonstrated in 1968 (Pinheiro et al. 1976). In fact, OROV is the only Orthobunyavirus that has currently been described in this mosquito species. Successful experimental transmission between infected and susceptible hamsters was achieved only in the presence of high viraemic levels in the vertebrate model (Hoch et al. 1987). However, this experiment was not conducted with natural host species of the virus, such as primates, sloths and birds and experimental reproduction of a viral disease in animal models frequently requires higher titres of the virus in the inoculum than those observed during natural infection (Pinheiro et al. 1981b).

Vector competence can vary according to the population of the mosquito present in different geographical regions. This behavioural change has already been shown for other arboviruses such as SLEV and West Nile virus, which are transmitted by*Cx*. *quinquefasciatus* (Reisen et al. 2005). Therefore, environmental factors can influence and change the competence of mosquitoes.

Despite of the maintenance of the specimens for more than 12 h on artificial feeding, the detection of segment S of OROV in *Cx. quinquefasciatus* captured in Cuiabá should be considered to result either from natural infection, especially in pools with nonengorged specimens or from blood meals in viraemic hosts in those pools with engorged specimens. Therefore, isolation of these viruses from nonengorged pools indicates that *Cx. quinquefasciatus *is a potential vector for OROV or other viruses from the Simbu serogroup in MT. Nevertheless, the importance of *Cx. quinquefasciatus* in the urban cycle of OROV is not completely understood or described in the literature. The involvement of *Cx. quinquefasciatus* with factors such as low viraemic titres in humans may explain the low number of cases identified in this study. Additional studies are necessary to elucidate the vector competence of*Cx. quinquefasciatus* for OROV or other orthobunyaviruses belonging to the Simbu serogroup in MT.

Specimens of *C. paraensis *or other Ceratopogonidae were not identified in this study. This result may be a result of either the absence of this species in the urban area of Cuiabá or the methodology used in this study. The chosen traps and period of capture might not be the most suitable for capturing biting midges (Santarém et al. 2010). Moreover, during epidemics, the isolation ratio of OROV in *C. paraensis* is considered extremely low (1:12.500). These data suggest that *C. paraensis* may be a low-efficiency vector and that other Culicidae may participate in the epidemiological cycle of OROV (LeDuc & Pinheiro 1986).

In the sampled sectors, *Cx. quinquefasciatus* presented a high potential for dispersion in different habitats. A study performed in Manaus demonstrated the temporal distribution of *Cx. quinquefasciatus*throughout the year, with peaks in January and April, during the rainy season (Barbosa et al. 2008).

The characterisation of new species within the OROV complex requires at least 14.1% and 20.9% aa divergence of the S and L segments, respectively (Ladner et al. 2014). Phylogenetic analysis indicates that the segment S OROV sequences obtained from humans and female *Cx*.*quinquefasciatus* pools in this study belong to the subgenotype Ia, the most frequently reported subgenotype in humans in Brazil and shows close proximity to sequences of OROV obtained from the cerebrospinal fluid of humans in Manaus (Bastos et al. 2012), from human blood in AC (Terzian et al. 2009), from humans, sloths (*B. tridactylus*), *Cx. quinquefasciatus* and*Ae.* (*Oc.) serratus *in PA, beyond the prototype OROV TRVL-9760 (Fig. 3) (Saeed et al. 2000, Vasconcelos et al. 2009).

The Orthobunyavirus sequences identified in humans in this study presented a high similarity with those identified in mosquitoes, indicating the same virus is circulating in both populations. This virus may have dispersed from the Amazon Region to neighbouring states and *Cx*.*quinquefasciatus* might be involved in the transmission cycle because the virus was identified in nonengorged pools and this species is considered a competent vector for OROV.

The segment S sequence obtained from sample CbaAR632 is very similar to and formed a cluster with the segment S sequence of OROV obtained from sloths (*B. tridactylus*) in PA in 1971 (Pinheiro et al. 1976) (Fig. 3). This pool, which contained two nonengorged *Cx*.*quinquefasciatus* females, presented the most divergent sequences. The aa substitution found in this sample was also observed in OROV sequences from subgenotype Ia obtained from sloths (*B. trydactilus)*in PA (Nunes et al. 2005). Moreover, primates and birds that are known to participate in the transmission cycle of OROV are commonly found in the preservation areas and parks of Cuiabá. Another aa substitution was found in this pool at position 339, which was reported previously only in human isolates of OROV from subgenotype IIa obtained in AP in 2009. A substitution at position 240 was observed previously in isolates from subgenotype IIIb obtained from *Callithrix* sp. in MG and from humans in MA, RO and AC and in Panamá (Vasconcelos et al. 2011). Although genotype I is considered the most stable, the mechanism of “boom and boost” evolution has also been reported for this genotype, resulting in emergence and posterior lineage replacement in the population (Zanotto et al. 1996).

A recent study suggests that nucleotide variations result from the selective pressure caused by the host in orthobunyaviruses. Therefore, sequences of the virus obtained from animals may have a different profile from those found in human and vector specimens. This characteristic is frequently observed within the M segment and is an important antigenic target that, due to the selective pressures and different geographical settings, can present high levels of nucleotide variation. Often, such variation can lead to a false interpretation of a reassortment (Tilston-Lunel et al. 2015). Because of its wide geographical distribution, viruses belonging to the Simbu serogroup present high levels of genetic diversity.

A phylogenetic study with species of the Simbu serogroup demonstrated that the three segments were consistent with their respective species with the exception of OROV, which presented various potential reassortment events (Ladner et al. 2014, Tilston-Lunel et al. 2015). In this study, it was not possible to obtain the sequences of the three segments due to the absence of viable biological samples from humans and the RNA extraction and sequencing methods selected for mosquito samples. However, there are limitations to the generation of new orthobunyaviruses by reassortment, such as the incompatibility of certain combinations, especially between distinct serogroups (Elliott 2014). Therefore, the detection of segment S of OROV suggests the possible circulation of this virus or a recombinant derivative of this virus in the state because, among orthobunyaviruses of the Simbu serogroup, OROV is the only species with recognised circulation in humans in Brazil.

Among the viruses belonging to the Simbu serogroup, Iquitos, Jatobal and Madre de Dios viruses are derived from OROV reassortment, belonging to the OROV serocomplex, and have been described in South America (Saeed et al. 2001, Aguilar et al. 2011,Ladner et al. 2014). OROV, Iquitos virus and Madre de Dios have been associated with human disease (Ladner et al. 2014). The only report of Jatobal virus, a reassortant with segment S from Peruvian strains of OROV, dates from 1984 in Brazil, when it was isolated from the blood of the South American coon (*Nasua nasua*) in PA (Saeed et al. 2001). The Iquitos and Madre de Dios viruses present segments S and L of OROV and M of another virus within the Simbu serogroup. The Iquitos virus was isolated from an outbreak that occurred only in Iquitos, Peru, in 1999 (Aguilar et al. 2011) and has never been described in Brazil. Likewise, Madre de Dios is a reassortant identified in humans only in Madre de Dios, Peru (Ladner et al. 2014). The Utinga virus, obtained from sloths (*B. tridactylus*), consists of a distinct species within the Simbu serogroup previously reported in Brazil, but never associated with human disease (Ladner et al. 2014).

Therefore, this is believed to be the first report of the circulation of an Orthobunyavirus with segment S of OROV in MT. Human cases may occur sporadically in the state, especially during dengue outbreaks. The similarity between the isolates identified in humans and *Cx. quinquefasciatus* in this study suggests the same virus is circulating in both vertebrate and invertebrate hosts. Increased deforestation, urbanisation and human and animal circulation among different Amazonian subregions may contribute to the dissemination of arboviruses in Brazil.
